# A quadricuspid aortic valve in an asymptomatic 40-year-old man: a case report

**DOI:** 10.1186/s13256-018-1755-3

**Published:** 2018-08-16

**Authors:** Giovanni Pasanisi, Gaia Mazzanti, Biagio Sassone

**Affiliations:** 1 Department of Emergency, Division of Cardiology, Delta Hospital, Azienda Unità Sanitaria Locale di Ferrara, Ferrara, Italy; 2 Department of Emergency, Division of Cardiology, SS.ma Annunziata Hospital, Azienda Unità Sanitaria Locale di Ferrara, Ferrara, Italy; 3 Unità Operativa di Cardiologia, Ospedale del Delta, Azienda Unità Sanitaria Locale di Ferrara, Via Valle Oppio 2, 44023 Lagosanto, Ferrara, Italy

**Keywords:** Quadricuspid aortic valve, Congenital cardiac defect

## Abstract

**Background:**

Integrated transthoracic and transesophageal echocardiography enables identification and characterization of a quadricuspid aortic valve anomaly.

**Case presentation:**

A totally asymptomatic 40-year-old white man was referred to our Division of Cardiology after accidental finding of a heart murmur. Transesophageal echocardiography detected a quadricuspid aortic valve characterized by four cusps of equal size and severe aortic valvular regurgitation, without any further anomalies. He underwent a successful aortic valve repair.

**Conclusions:**

Quadricuspid aortic valve anomaly is a rare congenital cardiac defect that can cause progressive valvular complications.

## Background

Quadricuspid aortic valve (QAV) is a rare congenital cardiac defect. It can be isolated or associated with other congenital cardiac abnormalities, and can cause progressive valvular regurgitation. We present a case of a 40-year-old man with isolated QAV, responsible for severe valve regurgitation, who underwent aortic valve surgery. The peculiarities of this case are that despite the severity of valvular disease at presentation, our patient was totally asymptomatic; aortic valve repair by tricuspidization technique was preferred to valve replacement, but data about the clinical approach in patients with such a rare presentation are still scarce.

## Case presentation

During a periodic visit to a health surveillance program, a heart murmur was found in a 40-year-old white man. He was employed as metalworker; he did not refer cardiovascular risk factors, had no significant medical history, did not consume drugs, and was totally asymptomatic. A cardiovascular examination did not show signs of congestive heart failure. His blood pressure was 130/55 mmHg. A standard electrocardiogram was normal.

**Fig. 1:**
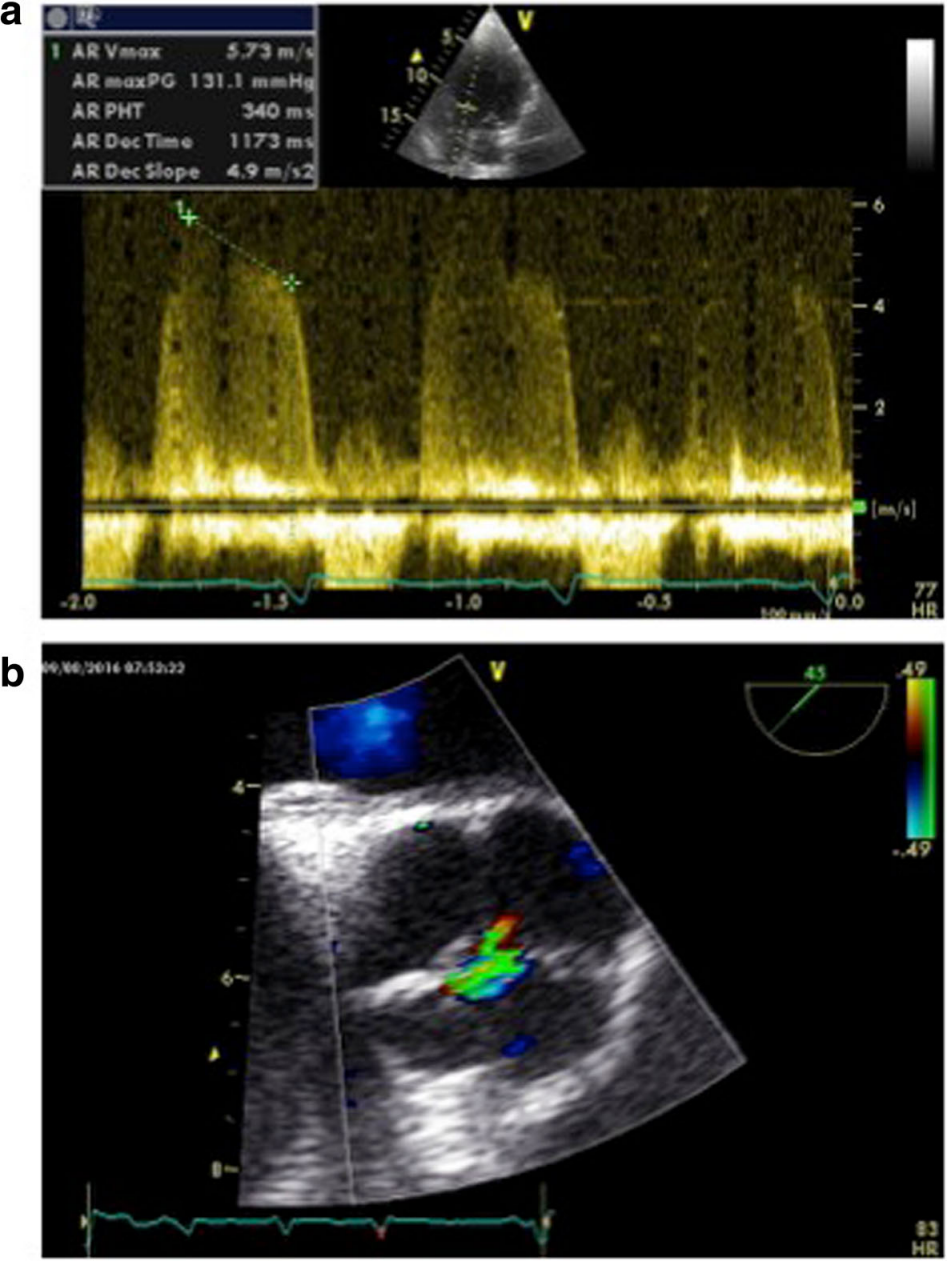
Echocardiography: severe aortic valve regurgitation (**a**) CW Doppler (**b**) Color Doppler

He was referred to our Cardiology Unit for transthoracic echocardiography. The transthoracic echocardiography showed: a left ventricle with normal dimension, wall thickness, and global and regional function; a severe aortic valvular regurgitation (Fig. 1a); and the suspicion of a dysmorphic valve that could not be better specified due to a poor acoustic window.

**Fig. 2:**
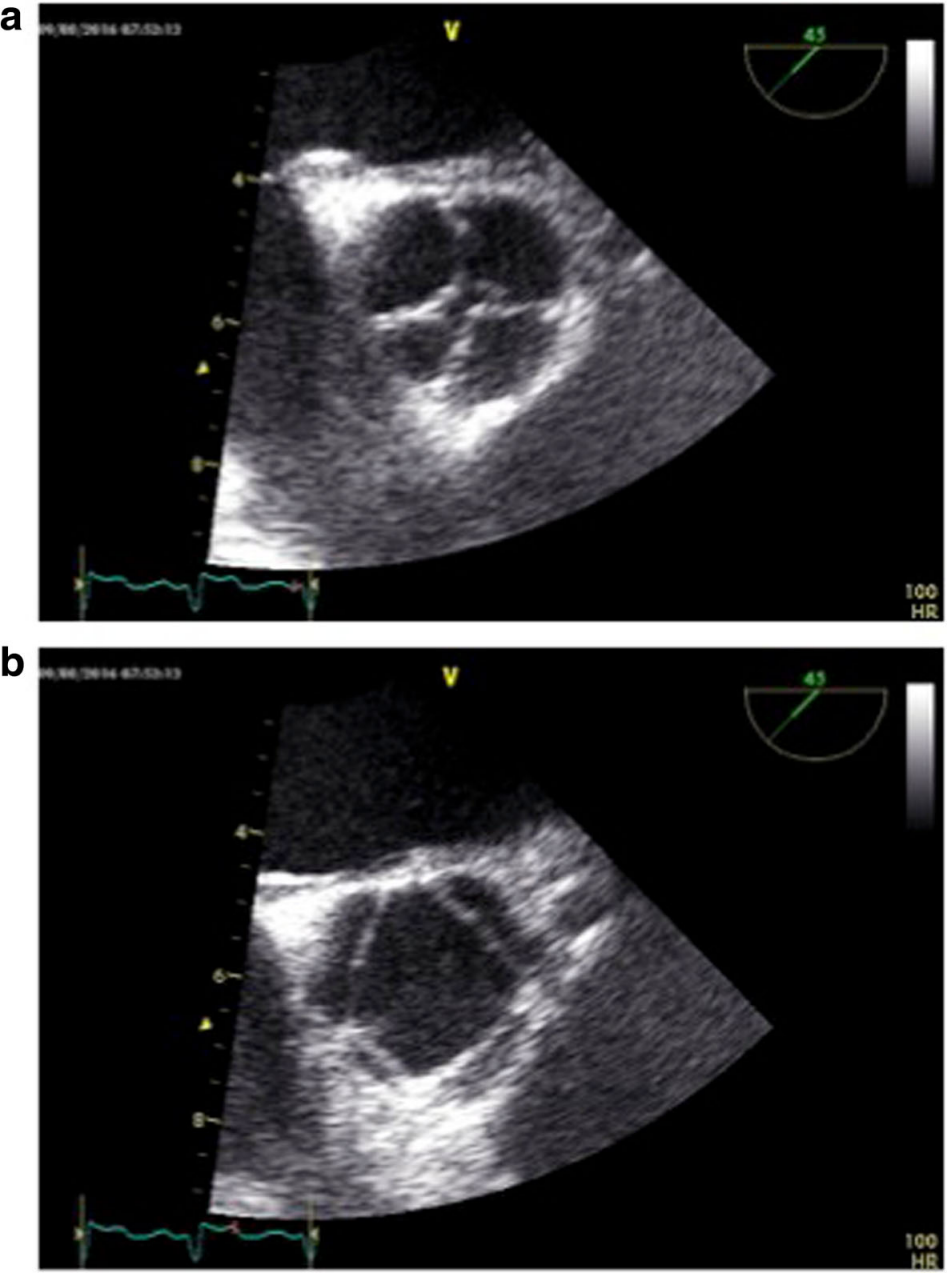
Transesophageal echocardiography: four cusps with “X” shape (**a**) Diastole (**b**) Systole

A transesophageal echocardiography was performed, which confirmed the presence of a severe aortic valvular regurgitation (Fig. 1b); the short axis view showed an aortic valve characterized by four cusps of almost equal size, with a regular profile and without degenerative modifications (Fig. 2). The examination did not reveal any further anomalies.

He was admitted to hospital. A cardiovascular examination did not show signs of congestive heart failure. His blood pressure was 130/50 mmHg and his temperature was 36.5 °C. Pulsus bisferiens was detected by palpating his carotid pulse (less evident in brachial pulse). No other physical abnormal findings were detected. A neurological examination was reported as normal. Routine blood tests were done, which revealed good blood count and good renal and hepatic functions. A stress test was not done. Before the cardiac valve surgery, our patient underwent coronary angiography that showed normal coronary arteries.

He underwent an aortic valve repair by tricuspidization technique, which was preferred to valve replacement because we did not want to expose our 40-year-old patient to valve-related risks across his lifespan.

He was treated with orally administered anticoagulant for 1 month after surgery. At 6-month follow-up visit he was asymptomatic and echocardiography detected only mild residual aortic regurgitation; he did not receive ongoing therapy.

## Discussion

We presented a case of QAV with peculiarities: QAV presented as an isolated defect and despite the severe valve regurgitation our patient was totally asymptomatic and underwent aortic valve surgery consisting of repair by tricuspidization technique.

**Fig. 3:**
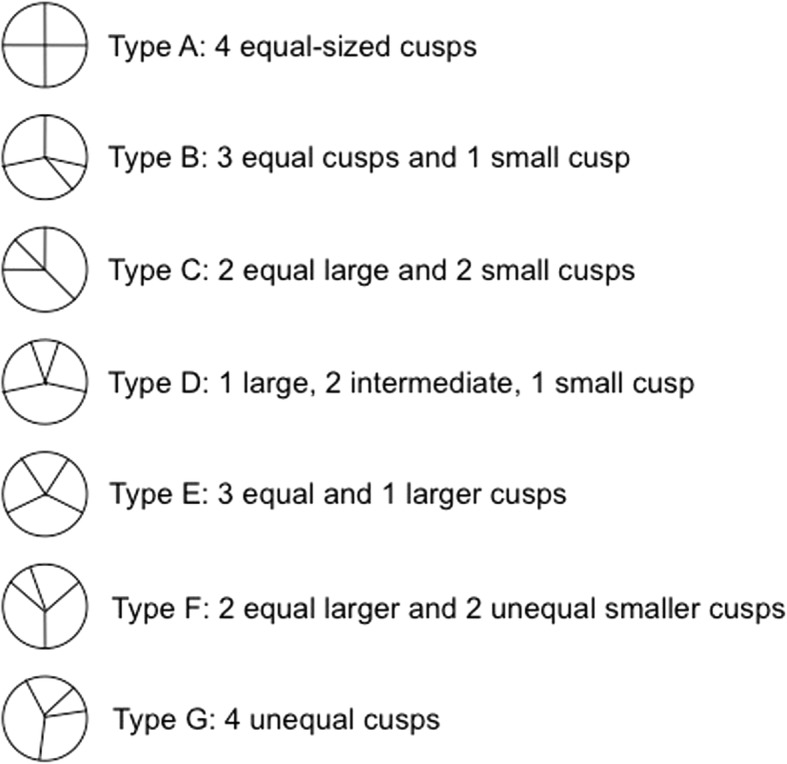
Hurwitz and Roberts’ classification of quadricuspid aortic valve [2]

QAV is a rare congenital cardiac defect with an incidence that varies from 0.006 [1] to 0.043% [2] and with a slight male predominance (male:female ratio 1.61:1) [3]. Aberrant cusp formation may represent abnormal fusion of the aorticopulmonary septum or abnormal mesenchymal proliferation in the truncus arteriosus [4]. According to the Hurwitz and Roberts’ classification, we distinguish seven anatomical variants of QAV depending on the dimensions of the cusps [2] (Fig. 3). Nakamura *et al.* provided a simplified classification into four types, based on the position of the supernumerary cusp [5].

QAV can appear as an isolated defect, although in 18% of cases it may be associated with other congenital anomalies such as: anomalous origin and/or coronary course, interatrial and/or interventricular septal defect, patent ductus arteriosus, mitral valve prolapse, Fallot tetralogy, partial atrioventricular canal, subaortic stenosis, non-obstructive hypertrophic cardiomyopathy, and ascending aortic aneurysm [1, 6]. QAV is most frequently associated with coronary abnormalities, which occur in 10% of cases. In the literature, a case is described of the sudden death of a young man with QAV, disclosed by necropsy. It was responsible for the adhesion of the left coronary cusp to the aortic bar, resulting in complete isolation of the orifice of the left coronary artery [7].

The most frequent complication of QAV is represented by aortic valve regurgitation, reported in 50–75% of cases [5, 8]; it is generally diagnosed in the fifth decade of life [9]. More rarely, the evolution of QAV into aortic valvular stenosis has been described [1]. Even rarer seems to be the occurrence of infective endocarditis [10]; infective endocarditis has been described mainly in the variants of QAV with asymmetric cusps, an anatomical situation that predisposes to greater flow turbulence that favors valve degenerative processes and consequently increases infectious risk. However, the latest guidelines on the management of infective endocarditis by the European Society of Cardiology do not recommend antibiotic prophylaxis in QAV [11].

The clinical manifestation of the QAV depends on the functional status of the valve and the presence of any associated anomalies. The most common symptoms are: palpitations, chest pain, dyspnea, fatigue, peripheral edema, and syncope [12].

Transthoracic and transesophageal echocardiography allow an easy diagnosis and can identify any complications or associated anomalies. In the short axis view for the aortic valve, in diastole, the closure of the cusps configures an “X” image (Fig. 2a) instead of the usual “Y” shape in the presence of a tricuspid valve.

Surgical indications are the presence of severe aortic stenosis, severe aortic regurgitation, or QAV with valvular dysfunction associated with other clinically significant abnormalities [1, 12]. The surgical options are replacement or valve repair according to tricuspidization techniques, bicuspidization, Ross procedure, or Manouguian’s operation [12]. Perioperative complications are generally rare and overall survival of patients with QAV, after 5 and 10 years of follow-up, is respectively 89.9% and 84.9% [1].

## Conclusions

A QAV anomaly is a rare congenital cardiac defect with a wide variety of clinical manifestations that depend on the functional status of the valve and the presence of any associated anomalies. Despite the severity of valvular disease at presentation, our patient was totally asymptomatic, thus representing the peculiarity of this case. Unfortunately, data about the clinical approach in patients with such a rare presentation are still scarce; however, our patient underwent a successful aortic valve repair.

## Abbreviation

QAV: Quadricuspid aortic valve
